# A global database on blowguns with links to geography and language

**DOI:** 10.1017/ehs.2025.10005

**Published:** 2025-08-27

**Authors:** Gabriel Aguirre-Fernández, Chiara Barbieri, Stephen C. Jett, Jorge D. Carrillo-Briceño, Rodrigo Cámara-Leret, Marcelo R. Sánchez-Villagra

**Affiliations:** 1 Department of Palaeontology, University of Zurich, Zurich, Switzerland; 2 Department of Life and Environmental Sciences, University of Cagliari, Cagliari, Italy; 3 Department of Evolutionary Biology and Environmental Studies, University of Zurich, Zurich, Switzerland; 4 Department of Biological and Agricultural Engineering, University of California, Davis, CA, USA; 5 Institute for Systematic and Evolutionary Botany, University of Zurich, Zurich, Switzerland

**Keywords:** Cultural evolution, weapon, curare, Austronesian, Caribe, hunting

## Abstract

The blowgun is a weapon that employs the force of breath for expelling a projectile and has been traditionally used for hunting and (occasionally) war. The use of blowguns extends to ancient times and is advantageous in dense-forest areas of South America and South East Asia. A classification system of blowgun types introduced in 1948 for South America is extended here. We assembled a global database that includes collection data and ethnographic accounts of blowgun types and other related features that were linked to available linguistic information. Our analyses show that geography explains the distribution of blowgun types to some degree, but within regions of the world it is possible to identify cultural connections. Darts are by far the most used projectiles and in combination with toxins (e.g. curare), these weapons reach their highest potential. A case study on the use of blowguns in groups of Austronesian language speakers shows clade-specific preferences across the tree. Our comprehensive database provides a general overview of large-scale patterns and suggests that incorporation of other related data (e.g. sights, mouthpieces, quivers) would enhance the understanding of fine-scale cultural patterns.

## Graphical abstract

**Figure:**
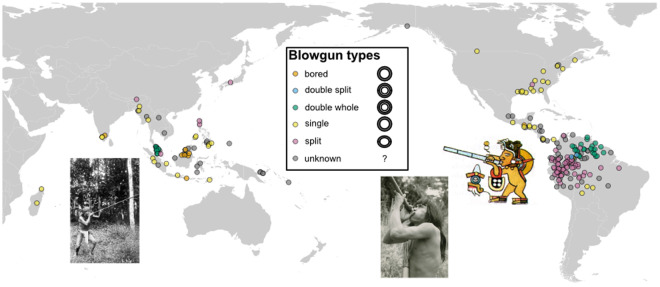

## Social media summary

The blowgun is a weapon with a long history and has been used in dense tropical forests as a main hunting tool. A global study of these weapons points to geographic and cultural links in different parts of the world.

## 1. Introduction

**Figure 1.**
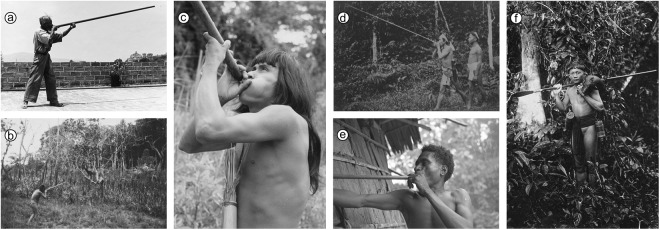
The traditional use of blowguns is famously prominent in South America (a–c) and South East Asia (d–f); (a) the last blowgun maker of Oaxaca, photographed by Sigvald Linné in 1948, CC-BY-NC-ND licence, obtained from http://kulturarvsdata.se/smvk-em/fotografi/2358495; (b) Piapoco man shooting a bird, photographed in the Colombian llanos by Gustaf Wilhelm Bolinder, public domain mark, obtained from http://kulturarvsdata.se/smvk-em/fotografi/2880155; (c) Achuar hunter photographed in Ecuador by Rolf D. Blomberg, CC-BY-NC-ND licence, obtained from http://kulturarvsdata.se/smvk-em/fotografi/25722074; (d) Senoi men in the Malay Peninsula, CC-BY-NC-ND licence, obtained from http://kulturarvsdata.se/smvk-em/fotografi/2741683; (e) Mejbrat man with a blowgun photographed by John-Erik Elmberg in the Bird’s Head Peninsula, Indonesia, CC-BY-NC-ND licence, obtained from http://kulturarvsdata.se/smvk-em/fotografi/25466495; (f) Kenyah hunter in Sarawak, Wellcome Collection, public domain mark, obtained from https://wellcomecollection.org/works/u656a9sg.

**Figure 2.**
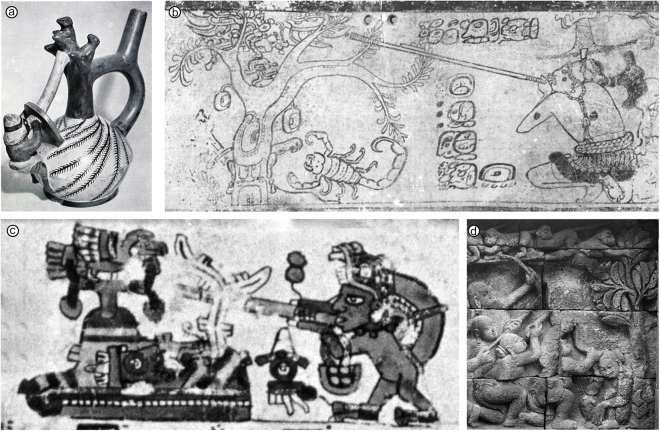
Iconographic evidence of ancient use of blowguns for hunting in: (a) a vessel of the Moche culture (100–700 CE) taken from Wasserman (1938), CC BY 4.0 license, obtained from https://bvpb.mcu.es/iberoamerica/es/consulta/registro.do?id=577492; (b) a Maya (250–900 CE) vase, photograph by Justin Kkerr, public domain, obtained from https://www.mayavase.com/; (c) the Codex Bodley (1300–1400 CE, Mixtec culture) by the Bodleian Libraries, University of Oxford, CC-BY-NC 4.0. Licence, obtained from https://digital.bodleian.ox.ac.uk/; and (d) a relief in Borobudur (800–900 CE, Java), photograph by Anandajoti Bhikkhu, CC BY-SA 3.0 licence, obtained from https://photodharma.net/. Note the depiction of pellets in (b) and (c).

The blowgun is a tubular weapon from which a projectile (usually a dart or a clay pellet) is propelled by the force of the human breath (Jett, 1970); this weapon excels for hunting birds and small mammals in dense tropical forests (Riley, 1952), but other reported less-frequent uses include warfare, sport and as a toy for children (Jett, 1970). Geographically, the traditional use of blowguns has a wide, but discontinuous distribution (Fig. 1): most ethnographic records come from the tropical zone on both sides of the Pacific – the Americas and South East Asia (Jett, 1991; Riley, 1954); other ethnographic records are from the south-east of the USA, Medieval Europe, parts of the Middle East, and Madagascar (Jett, 1970; White, 1960). The oldest archaeological records of blowguns (Fig. 2) date back to the fourth century in the Americas (e.g. Moche pottery vessels; Riley, 1954) and to the ninth century in South East Asia (reliefs at Borobudur, central Java; West, 2021). New World blowguns are most widely used and technologically diverse in the tropical forests of the Amazon and Orinoco basins. In the Old World, they are used in Peninsular Malaysia and on Sumatra, Java, Celebes, Timor, Mindanao, Luzon and many of the smaller neighbouring islands (Jett, 1970).

Yde (1948) distinguished four types of South American blowguns: Type I, a plain tube with a circular cross-section; Type II, two tubes, one inserted in the other; Type III, a single tube encased in a covering made of two halves held together by some sort of glue and a wrapping of bast or bark strips; and Type IV, composed of two halves of a split palm stem, glued together and wound with bark strips or bast fibres. Riley (1952) recognized three main types for a larger (New World) sample: the ‘two-tube weapon’, the ‘split-half composite’ and the simple ‘one-tube blowgun’; Riley (1952) also mentioned the presence of ‘intermediate forms’ between the ‘composite’ and ‘two-tube’ areas. Hypotheses on the construction and development of a world sample were discussed in depth in Jett (1970), the variation of types relies strongly on the availability of materials and the utility rank of the weapon. The single type (Type I, Table 1) is made of reed, bamboo or other plant stalk; the stems are basically hollow and a septum at each node of the stem separates adjacent internodes. Few species have internodes both long enough and of sufficiently small diameter to make ready-to-use blowguns; some solutions include punching out the septa with a rod of hard wood (or iron in modern times) and then polishing away the rough edges of the broken septa or joining (glueing and binding) internodes from which the nodes have been cut. The use of an outer tube approximately equal in length to the inner tube (type II, defined as double-whole in Table 1) stiffens the joint and protects the relatively delicate inner tube. The material of the outer tube can be bamboo or certain plants with soft central pith; palms are generally used as outer tubes in double-tube weapons in South America. The weapon here termed ‘double-split’ (Type III) in Table 1 is the rarest and is composed of a single tube encased in a covering made of two halves held together by glue and a wrapping of bast. The drilled wooden variety (termed bored in Table 1) is made by chopping or drilling a bore through a thick length of solid wood.

**Table 1.** Comparison of the main blowgun classification schemes

Yde (1948)	Riley (1952)	Jett (1970)	This paper
Type I – simple	Simple, one-tube	Single-tube, plant stems^a^	Single
Type II – double	Two-tube	Double-tube, one-piece outer	Double-whole
Type III – single, encased	Intermediate	Double-tube, one-piece inner	Double-split
Type IV – split	Split-half composite	Single-tube split-and-grooved	Split
NA	NA	Single-tube, bored^b^	Bored

a Reed or bamboo (internodes removed) and palm stem (pith pushed out).

b Wood.

Given the curious geographic distribution and antiquity of blowguns in South America and South East Asia, hypotheses on diffusion (e.g. Jett, 1970, 1991; Rivet, 1926; Stirling, 1938) or parallel invention (e.g. Riley, 1952; Yde, 1948) on a global scale have been discussed. This paper assembles data from first-hand observations in museum collections, published monographs (through literature searches and data collection from the eHRAF World Cultures database), and public online collection databases to provide the first computer- (and human-) readable world data set of blowguns. The database follows the FAIR principles (Wilkinson et al., 2016) and the recommendations for comparative cultural databases outlined in Slingerland et al. (2020). The association of cultural traits with the languages spoken by each society/ethnic group allows analyses under a phylogenetic framework, as outlined in Mace et al. (1994).

## 2. Methods

The primary units of analysis in this study are societies reported to use blowguns, which are here linked to languages (for ethnographic entries). Data were assembled from literature searches (including Jett’s active corpus of blowgun references), the eHRAF World Cultures database and collection-based research. The initial gathering was performed from the extensive works of Boglár (1950), Riley (1952, 1954) and Yde (1948). The search in eHRAF World Cultures was performed using the keyword ‘blowgun*’ (alternative tokens were also searched for, e.g., ‘blow gun*’, ‘blow-gun*’, ‘bodoquera’, ‘cerbatana’, ‘pucuna’, ‘sumpit’ and ‘blowpipe’, but the obtained data matched the more inclusive token ‘blowgun*’). The minimal information targeted included: ethnonym/culture name, blowgun type (when a given group used more than one type, this information is recorded in the database, but our analyses are restricted to the primary type), projectile type (mostly darts or pellets) and use (e.g. for hunting, for warfare, as a toy). Other fortuitously documented information includes the use of poison in darts and the type of prey (e.g. birds and mammals), local names given to the weapon, materials used for the weapon, projectile or toxin (if applicable), etc. The coverage (or completeness) of these data is generally low and would require further efforts in order to be conclusive. The data are also not mutually exclusive, but based on available information, for example, the category ‘poisoned darts’ in the field ‘ammunition’ is more exclusive than the category ‘darts’, which includes those that may be poisoned or not – the source simply does not provide sufficient detail. When dealing with multiple references for any given society, different ethnonyms were encountered and synonymized using Olson (1991) and the Ethnologue (Eberhard et al., 2024). To enhance information backtracking, we preserved the names as given to each group in the original publication (and provide the reference), being aware that some names were used as exonyms with derogatory connotations: society synonyms, when available, are supplied in a separate column (OSF data set). We also collected blowgun data from our own collection work in the following public repositories: Ethnographic Museum of the University of Zurich and Museum der Kulturen, Basel (in-person visits); American Museum of Natural History and Smithsonian National Museum of the American Indian, both in the USA (online catalogue searches).

The influential work by Yde (1948) provided ethnographic data linked to language families; based on this, we cross-checked and linked all ethnonyms with available language information using Glottolog 5.0 (Hammarström et al., 2024), a comprehensive catalogue of the world’s languages. Linguistic associations provided a more stable framework for comparisons when some groups were too coarsely defined (e.g. ‘Quechua’) and other groups too finely defined (e.g. Yabahana, a Yahuna subgroup); the refining of coarsely defined groups was made based on the positions plotted on the maps in the original publications and discarding overlaps among different sources. For example, the ‘Quechua’ entry of Yde (1948) was linked to the ‘Canelo’ entry of Riley (1952) based on the description of the locality in the former (Sarayacu River, Oriente, Ecuador), which roughly matches the map position of the latter (East of Ambato, in the Ecuadorian Amazon), which in turn is closest to the location of the ‘Northern Pastaza Quichua’ entry in Glottolog. There were two (of 286) cases of overlapping data (multiple entries referring to the same ethnographic group) with coding disagreements. In such cases, we provide notes on why we favoured one opinion over another; for example, Yde (1948) coded the blowguns of the Andoque (or Andoke) as ‘type I’, but Riley (1952) provided arguments to support the coding of ‘type IV’ as primary weapon, with children using the simpler ‘type I’ as a toy (see Table 1 for type terminology). It should also be noted that individuals within societies may speak more than one language, but the most representative language and/or that with geographic coordinates was prioritised and linked to a particular society. Comments on decision-making can be found in the field labelled ‘notes’ in the database. Geographic coordinates for almost all languages were obtained from Glottolog through the R package ‘Lingtypology’ (Moroz, 2017); the coordinates often represent the geographical centre-point of the area where the speakers live. For analyses, the following groups were lumped if referring to the language based on the fact that they speak the same language: subgroups of Baniwa (Karútana, Katapolítani, Kaua, Siusí and Yulámaua); Shawi (Cahuapana and Chayahuita); Cocama-Cocamilla (Cocama and Cocamilla); Cubeo (Cubeo and Bahuna); Madi (Wainamari and Yamamandi); Katukína-Kanamarí (Catuquina and Mangeroma); Macuna (Macuna, Buhagana); Matsés (Marawa and Mayoruna); Muniche (Muniche and Suchiche); Napo Lowland Quechua (Lowland Quechua and Quijo); Omagua (Omagua and Umava); Omurano (Mainas and Omurana); Pemon (Arecuna, Camaracoto and Taulipang); Shipibo-Conibo (Conebo, Sipibo and Setebo); Wapishana (Ataroi and Wapisiana). The database is publicly available here: https://doi.org/10.17605/OSF.IO/9Y7G3

## 3. Results

The original data set contains 286 societies; 20 are based on archaeological or colonial times records and 266 are based on ethnographic records. After data-quality checks and lumping of subgroups into comparable (language) units, the analyses reported below were performed for 231 society entries, three of which were based on archaeological records and 228 on ethnographic records. The societies in this data set can be linked to 227 languages of which 24 are isolates and the rest nest within 42 families (Table S1, supplement).

### 3.1. Blowguns

**Figure 3.**
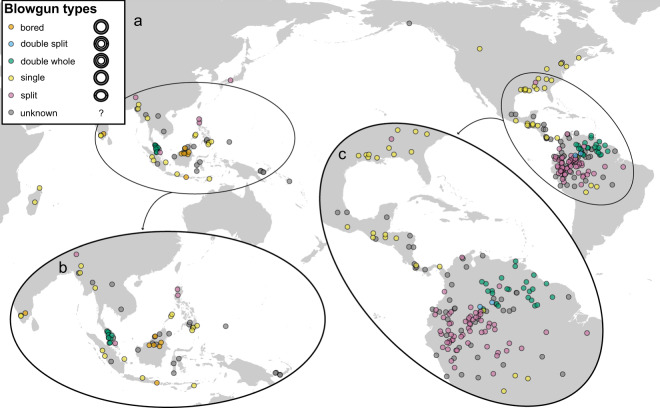
Distribution of blowgun types (see Table 1 for details) in the world sample (a). The two ‘hotspots’ for blowguns are located in South East Asia (b) and South America (c).

Of 155 records of blowgun types in the sample, the ‘split’ type is the most frequent, being represented in 67 society entries (43% of the total sample), followed by the ‘single’ type with 44 (28%), ‘double-whole’ with 34 entries (22%), and ‘bored’ with eight entries; entries for type ‘double-split’ are always related to groups that also have type ‘double-whole’ and are only reported for two groups in this sample. The following macro areas (as defined in Glottolog) are represented in the blowgun sample (Table S3, supplement): South America (*n* = 128), North America (*n* = 37), Papunesia (*n* = 37), Eurasia (*n* = 24) and Africa (*n* = 2) – these two entries are from Madagascar and can be culturally and linguistically linked to Austronesian speakers in insular South East Asia (Jett, 1970; Mitchell, 2020). We note that both the ‘single’ and the ‘split’ types are present in all four macro areas and, as mentioned above, are also the most frequent. The ‘single’ type has a more or less homogeneous distribution across the sample and in North America is used almost exclusively. On the other hand, the ‘split’ type is well represented in South America and poorly represented elsewhere. The ‘bored’ type is almost exclusive to Papunesian groups. The ‘double-whole’ type has a disjunct distribution involving South American and Eurasian groups. Our map plots (Fig. 3) show similar patterns to those in Yde (1948) and Riley (1952) for the Americas and those in Jett (1970, 1991) for the world (Fig. 3a). Geographic patterns in South East Asia suggest a preference for the ‘bored’ type in Borneo and for the ‘double-whole’ type in Malaysia (Fig. 3b); the ‘single’ blowgun has the widest distribution in South East Asia, and this is also true for the global sample (Fig. 3a) and the Americas. The geographic patterns in the Americas (Fig. 3c) show a clear preference for the ‘single’ type from Panama northwards (but also present in the southern-most areas of distribution in South America); a clear preference of the ‘double-whole’ type in the north-east of South America (Venezuela, Guyana, Suriname); and a preference of the ‘split’ type in the south-south-west area of the distribution area (mostly the Amazon Basin). In contrast to South East Asia (particularly Borneo), the ‘bored’ type is virtually absent in the Americas – the only reported case in this data set being that of the Jakaltek Maya (Ventura, 2003). The distribution of the eastern North American blowguns has been studied in some detail (Riley, 1952; Speck, 1938) and is discussed further below.

Two geographic ‘outliers’ found in our survey are the Eyak (autonym: DAXunhyuu) and the Blackfoot Confederacy; in both cases blowguns are only reported as used by children as toys. Birket-Smith and De Laguna (1938) reported small Eyak blowguns made of eagle or swan wing bones, whereas the Blackfoot use cow parsnip (*Heracleum maximum*) for the blowguns and chokecherries (*Prunus virginiana*) as projectiles; another variation reported for the Piegan (subgroup of Blackfoot) is the use of gumweed (*Grindelia squarrosa*) and water to kill insects (Hellson & Gaad, 1974).

**Figure 4.**
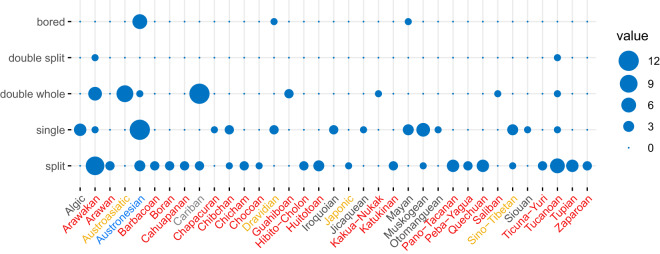
Balloon plot showing the number of groups and their associated blowgun types per language family. The text colours indicate the macro area (as defined in Glottolog): black, North America; red, South America; orange, Eurasia; blue, Papunesia; grey, the Chibchan family is distributed in the ‘Isthmo-Colombian area’ and therefore in both the North and South America macro areas.

As for the 42 language families linked to blowguns in this study (Table S1, supplement), 35 can be associated with a known type of blowgun (Fig. 4 and Table S2, supplement). The ‘split’ type is the most represented (recorded for 23 language families) and the preferred type for Arawakan speakers; the ‘single’ type is associated with 14 families and the preferred type for speakers of the Austronesian family; the ‘double-whole’ type is most popular in Cariban and Austroasiatic groups (to some degree, also Arawakan) – this disjunct pattern invites further study. The ‘double-split’ type is very restricted and only represented in two language groups: the Cubeo (a Tucanoan-speaking group) and the Baniwa (an Arawakan-speaking group); these are neighbouring groups in the Amazon near the boundaries between Brazil, Colombia and Venezuela (it should also be noted that groups using the ‘double-split’ also use the ‘double-whole’ type); the ‘bored’ type is essentially exclusive to Austronesian-speaking groups (Fig. 4) and representative of Borneo (Fig. 3).

**Figure 5.**
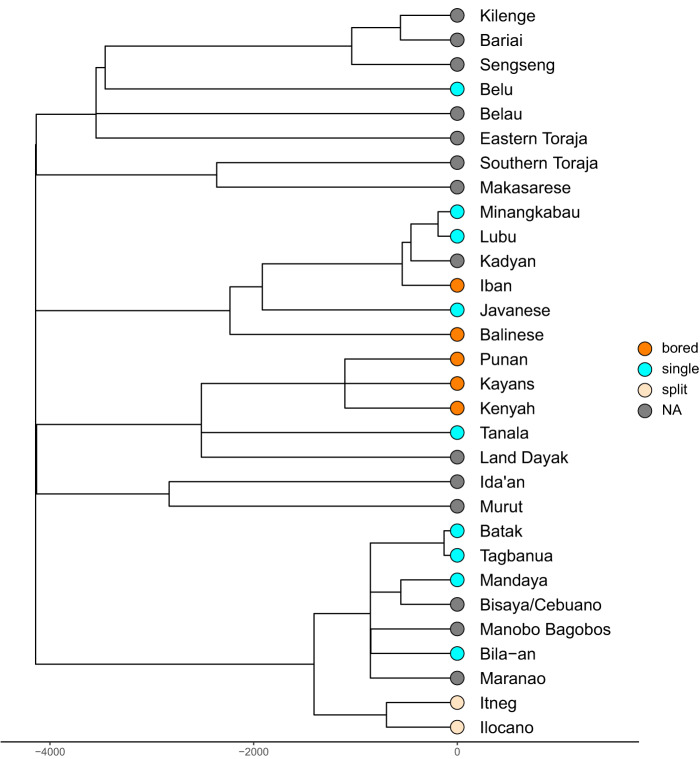
Tree of Austronesian languages and their blowgun representation.

The most represented linguistic group in this data set is the Austronesian family (Supplementary Table S1). This large language family was studied in the comprehensive work of Gray et al. (2009), who generated a time-calibrated phylogenetic Bayesian tree of 400 Austronesian languages. As a case study, 41 Austronesian-speaking societies in our data set were assigned to a second set of Glottolog proxies to maximize the matches with the existing phylogeny. Each society was associated with available dialects or closely related linguistic clades, or sister clades when the branch is not represented by any other society – therefore, respecting the relative position of the society in the phylogeny. This resulted in a subset of the Austronesian tree including 31 languages (or tree tips). We displayed the type of blowgun associated with each society/language in the tree (Fig. 5). Some linguistic branches display the same type of blowgun: this is the case for the Kayan-Murik (+Kenyah), who produce bored blowguns; the Greater Central Philippines (+Bilic), who produce single blowguns; and the Northern Luzon, who produce split blowguns. These correlations are particularly interesting for linguistically and related branches in the same region, the Philippines: here, Luzon speakers produce a characteristic blowgun type that is different than the one found in Bilic and Greater Central Philippines speakers, showing associations that need to be explored further. These observations are only descriptive, and should be corroborated with a more complete information on the type of blowgun for each society reported.

### 3.2. Projectiles and toxins

**Figure 6.**
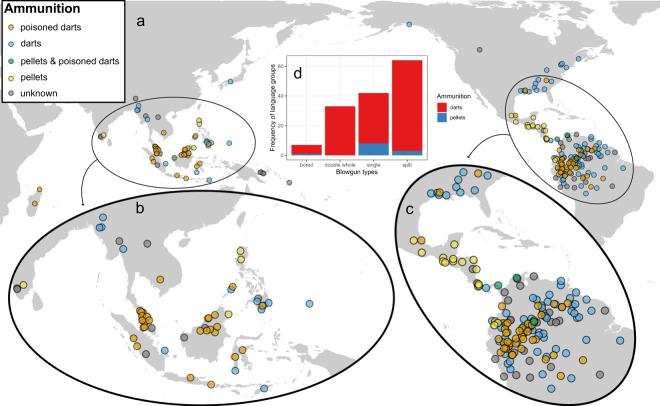
Map showing projectile types and the use of toxins in the world sample (a). Grey points represent unavailable data. Poison is only used in association with darts within this data set (not in pellets). The term ‘darts’ does not exclude an association with the use of poison, but may rather reflect a lack of information. The eastern USA is generally believed to use darts without toxins, but this has never been systematically studied and is therefore regarded as ambiguous. Our results show that some North American groups are reported to use toxins. The two ‘hotspots’ for blowguns are located in South East Asia (b) and South America (c). Darts are much more prevalent than pellets and pellets are more strongly associated with the ‘single’ type (d).

Darts are by far the most frequently used projectiles (Fig. 6), with 155 entries; followed by pellets, including seeds (20 entries). Only three societies use both: the Kogi speakers (autonym Kaugiañ), the Kuna speakers (autonym Duleigaiya) and the Tucano speakers (autonym Dasea ye). Pellets are depicted in the oldest evidence of blowguns for Nuclear America, including iconography of the Moche, Aztecs and Maya (Fig. 2). These reliefs and vase drawings represent bird hunting, as still done by the Jakaltek Maya (Ventura, 2003). Our available data show that pellets are associated more strongly to the ‘single’ blowgun type, are almost exclusively used for hunting birds and are almost exclusively owned by groups living to the north of the Equator and to the south of the Tropic of Cancer (0 to 23 °N). The use of darts is widely distributed and mostly associated with hunting of arboreal mammals. Direct information on targetted prey and efficiency of blowguns has been very poorly explored, but see the discussion for more detail on the few exceptions. As stated in the results, the category ‘poisoned darts’ is basically a subset of the category ‘darts’ for which we explicitly know the association between darts and use of poison; it is quite likely that the number of groups using poison in their darts is greater, but we were unable to recover such detail in our survey.

Mentions of the use of toxins were found for 79 societies in this data set (Fig. 6) – these are mostly generic (e.g. ‘poisoned darts’), but some mention the use of curare or frog poison. In any case, the practice of using frogs for blowgun dart poisoning is very rare and Daly et al. (1978) were able to determine with certainty that only the Nonamá Chocó and Emberá Chocó of western Colombia have employed frogs for poisoning. The plant-based toxic substances used to tip blowgun darts in the Amazon are generally known as ‘curare’. A literature review of 505 Amazonian ethnobotanical references (Cámara-Leret et al., 2024) indicates that at least 196 plant species in 50 families and 104 genera are used by indigenous communities in Amazonia to make curare. The most diverse plant families are the Loganiaceae (35 species), Menispermaceae (30) and Annonaceae (10). For 23 plant families, only one species has been recorded. Plant genera with at least 10 curare species include *Strychnos* (35 species), *Piper* (11) and *Abuta* (10 species). For 77 genera, only one species has been reported. The most common plant parts used to manufacture curare are the bark (*n* = 86 species), root (*n* = 48) or stem (*n* = 32). So far, the surveyed literature contains curare reports from 56 of the ca. 300 Indigenous groups that live in Amazonia. In short, these data suggest that many plant families, genera and Indigenous cultures are still imperfectly known and merit further research. Using the number of Indigenous groups as a proxy for geographic range of curare, we find that the most widely used species are *Curarea tecunarum* (12 Indigenous groups), *Abuta grandifolia* (11) and *Strychnos guianensis* (11). On average, Indigenous groups know 6 species (min: 5, max: 33, sd: 7). The Cofan have reported the highest number of curare species (33), followed by the Ticuna (30) and the Barasana and Kitchwa del Oriente (both 20). By contrast, 14 groups report only one species. It is remarkable that 104 species are represented by but a single Indigenous group. The composition of curares shows strong geographical and cultural associations. For example, *C. tecunarum* is only reported by western Amazonian Indigenous groups; *A. grandifolia* is reported by 10 cultures from western Amazonia and by only one from the Guianas (Wayana); and *S. guianensis* is reported by five western Amazonian cultures and by six eastern Amazonian cultures. As stated above, many curare species are only reported by a single Indigenous group, meaning that geographically proximate cultures utilize different species.

## 4. Discussion and conclusion

This work provides a general overview of large-scale patterns in the use of blowguns across the globe and complements detailed descriptive ethnographic work in a format more amenable to quantitative analyses. The geographic distribution of blowguns is clearly non-random, but cannot be explained by a single factor. Non-environmental historical factors such as migrations no doubt played significant roles as well but are not explored here. The archaeological record shows that blowguns appeared in both the Old and the New World before the age of discovery. Discussions on long-distance cultural diffusion across the Pacific are common in fundamental publications on blowguns (Jett, 1970; Riley, 1952; West, 2021) and in other contexts (Jett, 2017); for example, similarities in panpipe features (Aguirre-Fernández et al., 2020; Sachs, 1940), the possible introduction of Polynesian chickens into Chile (Storey et al., 2007), patterns of diffusion of the sweet potato into Oceania (Roullier et al., 2013) and the presence of Austronesian genes in some Amazonian Native American societies (Skoglund et al., 2015). Parallels in myth cults and gender relations (Gregor & Tuzin, 2001) and recent work on linguistics has also conjectured a deep-time link among languages in those regions (Bickel, 2020; Nichols, 1992).

Further understanding can be achieved by enriching this data set with information on variables with low representation (e.g. materials, prey, environment, weapon name in language of origin). Availability of materials plays a role, as noted by Friederici (1911) and Nordenskiöld (1924) early on: the two main clusters in South America involve the ‘double-whole’ made of *Arundinaria* in the north-east and the ‘split’ type made of palm in the western part (e.g. *Iriartella setigera* and *Bactris maraja* are used by the Nukak; Politis, 2007). Note that there was long-distance trade in some materials involved in the blowgun complex. The botany of the production of blowguns is a subject of further study, one for which information is available in numerous ethnographic accounts (Jett, 1970; Nordenskiöld, 1924), but needs systematic database work. The most used species are *Kinabaluchloa wrayi* for the Old World and *Arthrostylidium* spp or *Arundinaria* spp for the New World (Jett, 1970). There is little documentation regarding which kinds of prey are specifically targeted and the success rate of hunting using blowguns. Data collected by Yost and Kelley (1983) for the Waorani indicate that 64% of the kills (relating to animals weighing more than 10 kg) were performed using a blowgun, followed by shotguns (33%) and spears (3%); they also listed 32 species that were killed using blowguns. The top 5 prey are: woolly monkey (*Lagothrix lagotricha*), Cuvier’s toucan (*Ramphastos cuvieri*), Venezuelan red howler (*Alouatta seniculus*), Spix’s guan (*Penelope jacquacu*) and northern Amazon red squirrel (*Sciurus igniventris*). Marshall et al. (2006) concluded that the best predictor for orangutan density in the forests of Kalimatan is not logging intensity, but distance to the nearest (blowgun) hunting village. Again, more data on kinds of prey for each society would provide valuable information for further analyses. Blowgun ‘toys’ (for children, particularly boys) are sometimes referred to in ethnographies (including 11 societies in our data set) and can be interpreted as early training. The use of these ‘toys’ (i.e. miniature versions, quickly made and with easy-to-find materials) have been reported for other weapons (e.g. spear throwers, bow and arrow) and their use starts early in life: for bow and arrow at age 3 or even before (Kamp & Whittaker, 2020). In a few cases, the toy is present in the absence of a ‘practical adult version’, which has been interpreted as a relict of a weapon that fell in disuse (Jett, 1970).

Although there are clear preferences for blowgun types and use of projectiles among different areas and/or groups, their construction and combination (also with other aspects, e.g. sights, mouthpieces, projectiles, quiver types) are important to an understanding of finer patterns than those shown here. Mouthpieces of several shapes and materials have been recorded (Jett, 1970) and may provide key information for comparisons, but as noted in Yde (1948), information on mouthpieces is very irregular in the literature and they are often missing from the specimens in collections; therefore, a systematic effort to document them would be very valuable. Little-known materials and methods for the production of blowguns and projectiles need to be put into context – for example, the glue darts used by the Nukak (Politis, 2007) employed to hunt certain birds or the use of bones instead of plant material for the construction of ‘blowguns’ (?) by the Eyak (Birket-Smith & De Laguna, 1938). When available, the ‘operational sequence’ (e.g. as for the Nukak in Politis, 2007) may provide a framework for even finer studies, although potential pitfalls need to be evaluated (Bar-Yosef & Van Peer, 2009). Better understanding of the time component would surely disentangle some of the most debated questions on this topic; for example, there is the hypothesis that blowguns were insignificant until curare started being used, which resulted in the invention and spread of the ‘split’ type, the most popular in South America (Nordenskiöld, 1924).

## Supporting information

Aguirre-Fernández et al. supplementary material Aguirre-Fernández et al. supplementary material

## Data Availability

The data associated with this research are available at the following OSF link: https://doi.org/10.17605/OSF.IO/9Y7G3

## Supplementary material

The supplementary material for this article can be found at https://doi.org/10.1017/ehs.2025.10005

## Research transparency and reproducibility interest

All data used in this study are provided within the manuscript, the supplementary material and the OSF link: https://osf.io/9y7g3/?view_only=0268b4045c6f446ca1931d578709f1bb
