# Supplementary material for: A global database on blowguns with links to geography and language
Source: Evol Hum Sci. 2025 Aug 27;7:e26. doi: 10.1017/ehs.2025.10005 (PMC12516597; doi:10.1017/ehs.2025.10005)
Supplement: Aguirre-Fernández et al. supplementary material [file S2513843X25100054sup001.pdf]

Supplement to:

A global database on blowguns with links to geography and language

Gabriel Aguirre-Fernández[1], Chiara Barbieri[2, 3], Stephen C. Jett[4], Jorge D. Carrillo-Briceño[1], Rodrigo Cámara-Leret[5], and Marcelo R. Sánchez-Villagra[1]

[1]Department of Palaeontology, University of Zurich, Switzerland
[2]Department of Life and Environmental Sciences, University of Cagliari, Cagliari, Italy
[3]Department of Evolutionary Biology and Environmental Studies, University of Zurich, Zurich, Switzerland
[4]Department of Biological and Agricultural Engineering, University of California, Davis, USA (Emeritus)
[5]Institute for Systematic and Evolutionary Botany, University of Zurich, Switzerland

Table S1: Number of languages per family

	Language family	Language count
1	Austronesian	41
2	Arawakan	26
3	Isolate or unknown	24
4	Cariban	15
5	Austroasiatic	11
6	Tucanoan	11
7	Chibchan	9
8	Pano-Tacanan	8
9	Mayan	6
10	Muskogean	6
11	Sino-Tibetan	6
12	Tupian	6
13	Quechuan	5
14	Algic	4
15	Huitotoan	4
16	Iroquoian	4
17	Arawan	3
18	Chocoan	3
19	Dravidian	3
20	Misumalpan	3
21	Zaparoan	3
22	Barbacoan	2
23	Boran	2
24	Cahuapanan	2
25	Chicham	2
26	Guahiboan	2
27	Hibito-Cholon	2
28	Katukinan	2
29	Peba-Yagua	2
30	Ticuna-Yuri	2
31	Athabaskan-Eyak-Tlingit	1
32	Chapacuran	1
33	Harakmbut	1
34	Japonic	1
35	Jicaquean	1
36	Kakua-Nukak	1
37	Otomanguean	1
38	Saliban	1
39	Siouan	1
40	Tai-Kadai	1
42	Uto-Aztecan	1
43	Yanomamic	1

Table S2: Contingency table showing frequency of blowgun types per language, grouped into language families.

	bored	double split	double whole	single	split
Algic	0	0	0	4	0
Arawakan	0	1	5	1	10
Arawan	0	0	0	0	2
Austroasiatic	0	0	8	0	0
Austronesian	6	0	1	12	3
Barbacoan	0	0	0	0	2
Boran	0	0	0	0	2
Cahuapanan	0	0	0	0	2
Cariban	0	0	12	0	2
Chapacuran	0	0	0	1	0
Chibchan	0	0	0	2	1
Chicham	0	0	0	0	2
Chocoan	0	0	0	0	1
Dravidian	1	0	0	2	0
Guahiboan	0	0	2	0	0
Hibito-Cholon	0	0	0	0	2
Huitotoan	0	0	0	0	3
Iroquoian	0	0	0	2	0
Japonic	0	0	0	0	1
Jicaquean	0	0	0	1	0
Kakua-Nukak	0	0	1	0	0
Katukinan	0	0	0	0	2
Mayan	1	0	0	3	0
Muskogean	0	0	0	5	1
Otomanguean	0	0	0	1	0
Pano-Tacanan	0	0	0	0	4
Peba-Yagua	0	0	0	0	2
Quechuan	0	0	0	0	4
Saliban	0	0	1	0	0
Sino-Tibetan	0	0	0	3	1
Siouan	0	0	0	1	0
Ticuna-Yuri	0	0	0	0	2
Tucanoan	0	1	1	1	6
Tupian	0	0	0	0	4
Zaparoan	0	0	0	0	2

Table S3: Contingency table showing frequency of blowgun types per macro-area.

	bored	double split	double whole	single	split
Africa	0	0	0	2	0
Eurasia	1	0	9	5	3
North America	1	0	0	22	1
Papunesia	6	0	0	10	2
South America	0	2	25	5	61

Key to principal blowgun features for a world survey: guide for collection managers

1. Blowgun type
    - simple hollow-plant-stem tube of bamboo (cane) or other natural material (perhaps with pith pushed out)
    - two hollow-plant-stem tubes, one inside the other
    - length of solid wood, split and grooved
    - length of solid wood (whole), bored with chisel
    - other (specify)
2. Length of blowgun (in cm)
3. Name (multiple names possible)
4. Dart type
    - fiber wadding (specify fiber; container for fiber?)
    - pith stop
    - other type of dart (specify)
    - clay pellet
    - pellet of other material, including seeds (specify)
5. Quiver type
    - bamboo section; cap? engraving?
    - basketry; pitch-smeared? other characteristics
    - wood; describe; belt hook?
    - palm leaf
    - other
6. Poison type
    - *Strychnos toxifera*
    - *Strychnos strychnos*
    - upas (ipoh, *Antiaris toxicaria*[?])
    - American cardiac poison
    - snake venom
    - frog toxin
    - other (specify)

Note other facts related to the blowgun, such as treatment of the bore (polishing; tapering bore; curving compensation for weight of gun), presence of spear point attached to gun, sight, ritual surrounding gun and especially poison making, poison antidotes, wrapping and other covering on the blowgun, etc.
